# Unusual Effects of Quinine

**Published:** 1878-12

**Authors:** Frederick D. Lente


### UNUSUAL EFFECTS OF QUININE.

By FREDERICK D. LENTE, M.D.

In the "Reports on the Progress of Medicine" in the New York *Medical Journal* for August, 1878, is a notice of several cases, in which, after the administration of quinine in moderate doses, a profuse exanthem appeared, which lasted several days, and was followed by desquamation, lasting from two to three weeks. Two of the cases were observed by Prof. Koebner, of Bresleau. In one case there was a repetition of the phenomena after a repetition of the dose. Dr. Ricklin, who reports the cases in the *Gazette Medicale*, was able to discover only four analogous cases in medical literature. But the "Report" states that Dr. Pflueger, of Berne, found the eruption to appear after the administration of the decoction of cinchona as well as after sulphate of quinine. In one of the cases the eruption resembled *urticaria*, but in this case the desquamation lasted three weeks. In all the cases the general symptoms were severe and even alarming; in one, that of a physician who took a gramme of quinine for facial neuralgia, "there was intense fever, delirium, dyspnœa, and all the signs of pulmonary congestion." Dr. Ricklin considers these cases important, "especially in view of the large number of pretended relapses of febrile exanthemata, particularly of scarlatina, which have lately been recorded."

Serious symptoms following the use of quinine are so rare (though not unfrequently alleged by non-professional persons, especially those under the care of homœpathic physicians) that the writer deems it of sufficient interest in this connection to refer to a few cases which came under his own observation a few years ago while practicing at Cold Spring.

A delicate laundress in the employ of a neighbor of the writer required a tonic, and when he proposed quinine she stated that it had produced dangerous symptoms when administered on a former occasion. One of the ordinary elixirs of calisaya was accordingly prescribed, and in a diminished dose. On his return home after an absence of some hours, his associate, Dr. G. W. Murdock, informed him that the first dose produced very unpleasant symptoms, and that, after the second, he was summoned and found her in a comatose state, with cold extremities, hot head, and symptoms of cerebral congestion. He combated these alarming manifestations with vigorous measures, including cupping of the temples, hot stimulating pediluvia, etc., and was obliged to remain with her four hours before she was out of danger. She afterwards informed the writer that a sister had been similarly, but not so seriously, affected by a small dose of quinine.

Soon after this occurrence, the writer prescribed a moderate dose of quinine for a female domestic in the family of a gentleman boarding at Garrison's. He then proceeded to West Point, and some hours after, on his return, met a messenger, who had been sent to find him, with the message that the girl had been taken suddenly very ill. He found her recovering from the attack, which had presented symptoms similar to those above narrated, but in a much milder degree.

A lady patient of the writer's, in the same neighborhood, informed him that quinine always weakened instead of strengthening her. He tried the experiment with small doses without her suspecting it, but she soon detected it by the peculiar prostration which it produced. He then tried the citrate of iron and quinine, but even this was detected in the same manner. At about the same time a case occurred in the practice of a medical acquaintance on Staten Island. A lady patient informed him, when he proposed to administer quinine, that it had always produced alarming symptoms; he, however, rather ridiculed the idea, and said he would give her a small dose and remain with her to observe its effect. He did so, and soon had reason to regret his experiment, as he was himself alarmed at the symptoms. What they were the writer did not learn, as the case was related to him by a friend of the patient.

These cases all occurred within a twelvemonth, and never before or since, in a large quinine experience, and with doses of every size (and he has recorded cases where an ounce was taken, *vide* H. C. Wood's *Mat. Med.*), has the writer met with a similar case.

Apropos of the eruptive phenomena, it may be well to state that during the prevalence of malarious fever in Cold Spring, some years ago, the attacks, in several instances, were ushered in by a profuse and typical *urticaria*, which disappeared quite promptly, whether in consequence of the full doses of quinine administered, or the full development of the febrile paroxysm it is impossible to say. In one female patient, who had repeated attacks of the fever, the latter could always be predicted by the appearance of the eruption. Last summer a gentleman at Saratoga Springs, a healthy man, applied to the writer for relief from a most troublesome attack of acute urticaria,. for which, on close inquiry, he could ascribe no cause. He had not been exposed to miasmatic influences, as far as he was aware. He had taken a few powders prescribed by a homœopathic physician, but without any relief. His legs were considerably swollen, his face somewhat so. General baths of tepid water, impregnated with borax and carbonate of soda, were prescribed, which relieved the urgent symptoms. In view of the usual tardiness of the ordinary treatment of these cases, and also believing that we must look to the nervous system for the pathology of the disease, quinine in doses of ten grains was prescribed, to be repeated *pro re nata.* This was effectual. Relief would follow and continue for some hours. Then the eruption would commence to re-appear, and, on a repetition of the dose, would soon commence to decline. After five or six doses he was entirely relieved. He continued well when last seen a couple of weeks later. There was no desquamation.

It is noteworthy that all the writer's cases, in which severe or unpleasant symptoms followed the ingestion of quinine, occurred in the person of females. The sex is mentioned in only two of Dr. Ricklin's cases. One was a male, the other a female. Should quinine prove to be a remedy for urticaria, it might, in the light of these histories, be taken as an illustration of the doctrine of *similia similibus curantur.*

Since writing the above, and on taking up a late number of the Cincinnati *Lancet and Clinic*, it is noticed that Dr. G. W. H. Kemper asks the question: "Does sulphate of cinchonidia cause urticaria and puffiness?" He states that both he and his partner have observed the unusual number of cases of urticaria, especially among children attacked with malarial fever. It is quite common, he states, for families to use the sulphate of cinchonidia for fevers, and quite as common to be consulted by them for hives, nettle-rash, etc., also puffiness of face, eyelids, and extremities, independent of the urticaria. He says: "I have seen nothing on the subject, but have arrived at the conclusion that the sulphate of cinchonidia is the cause of the urticaria." He says, however, that he has known an individual, after taking sulphate of quinine, always to suffer from an erythema to such an extent that general desquamation followed. In this case, cinchonidia had the same effect. He adds: "I would be glad to hear from members of the profession upon this interesting and practical question. I formerly thought that *malaria* caused urticaria, but have abandoned that idea."

In the last edition of Ringer's *Hand-book*, it is also noticed that he says: "Quinine, in one of my patients, always brings out a uniform red rash over the whole surface of the body. * * * Desquamation, as free as after a sharp attack of scarlet fever, always follows the rash." He states that "it is well known that quinine can produce urticaria."—*New York Medical Record.*

